# Supplementary material for: Dynamic analysis and control of a rice-pest system under transcritical bifurcations
Source: PeerJ. 2023 Oct 10;11:e16083. doi: 10.7717/peerj.16083 (PMC10573297; doi:10.7717/peerj.16083)
Supplement: Supplemental Information 1 [file peerj-11-16083-s001.pdf]

Supplemental Information 1. Modelling of the rice-pest dynamic system and its biological control

Supplemental Information 1.1 – Assumption and model formulation of the rice-pest dynamic system

Let $x_1(t)$ be the annual production of rice per unit area (hectare) and $x_2(t)$ be the corresponding pest species population at a time *t* where the annual production of rice acts as the prey population, and the corresponding pest species population acts as the predator population. In this regard, the pest population consumes and damages the rice growth which declines the annual production of rice. The relationships between the production of rice and the density of pests are as follows (*Chunyan & Daqing, 2013*; *Milligan et al., 2016*):

(i) At the initial state, let the reproduction rate of rice be α_1. For the consumption of rice by the pest population, a part of the annual production of rice is lost which let's β_1 be the loss rate of $x_1(t)$ due to the consumption of $x_2(t)$. Then the reproduction rate of rice can be represented by

$$\frac{d}{dt}x_1(t) = \left(\alpha_1 - \beta_1 x_2(t)\right)x_1(t) \tag{S1}$$

where $\beta_1 x_1(t) x_2(t)$ presents the annual loss of rice due to pests.

(ii) On the other hand, the pest population in the system depends on rice for feeding. Therefore, the pest population proportionally increases with the increasing rice production. Let β_2 be the energy gain rate of pest population by consuming rice. Again, since the pest population depends on the production of rice for feeding, their growth and population number will be reduced when the production of rice declines. Therefore, α_2 is taken as the decline rate of the pest population proportionally with the decline of rice production. Then the growth rate of the pest population can be represented by

$$\frac{d}{dt}x_2(t) = \left(\beta_2 x_1(t) - \alpha_2\right)x_2(t) \tag{S2}$$

where $\beta_2 x_1(t) x_2(t)$ presents the annual consumption of rice by the pest population.

The rice-pest system can be written in terms of a pair of Nonlinear Ordinary Differential Equations (NODEs) in form of a Lotka-Volterra model:

$$\begin{cases} \frac{d}{dt}x_1(t) = \left(\alpha_1 - \beta_1 x_2(t)\right)x_1(t) \\ \frac{d}{dt}x_2(t) = \left(\beta_2 x_1(t) - \alpha_2\right)x_2(t) \\ x(t) = (x_1(t), x_2(t)), \quad \forall t \\ x(t) = \left(x_{10},\, x_{20}\right), \; when \quad t = 0 \end{cases} \tag{S3}$$

where $x_1(t)$ and $x_2(t)$ respectively present the annual production of rice per unit area and the density of the rice pests at time *t*, and x_{10} and x_{20} respectively show the initial conditions of $x_1(t)$ and $x_2(t)$ i.e., the values of $x_2(t)$ and $x_2(t)$ at $t = 0$.

The production rate of rice is not damaged only by the attack of pests, but it may also be damaged owing to environmental impacts such as drought, global warming, etc. Similarly, the pest population may be reduced due to natural causes such as floods, droughts, etc. Therefore, two parameters i.e., d_1 (decrease rate of rice production due to natural causes not related to the pest population), and d_2 (death rate of pest population due to natural causes not related to the deficiency of rice) need to be considered in the model as well:

$$\begin{cases} \frac{d}{dt}x_1(t) = \left(\alpha_1 - \beta_1 x_2(t)\right)x_1(t) - d_1 x_1^{\,2}(t) \\ \frac{d}{dt}x_2(t) = \left(\beta_2 x_1(t) - \alpha_2\right)x_2(t) - d_2 x_2^{\,2}(t) \\ x(t) = (x_1(t), x_2(t)), \quad \forall t \\ x(t) = \left(x_{10},\ x_{20}\right),\ when \quad t = 0 \end{cases} \tag{S4}$$

Here, the term $d_1 x_1^{\,2}(t)$ presents the decrease rate due to intraspecific competition in species $x_1(t)$ due to natural causes that are not related to $x_2(t)$, e.g., viral infections, droughts, or floods (*Bazykin, 1976*; *Liu & Jiang, 2021*). Similarly, the term $d_2 x_2^{\,2}(t)$ shows the intraspecific competition between $x_2(t)$ due to natural causes that are not related to $x_1(t)$, e.g., viral infection and heavy rains (*Bazykin, 1976*; *Yang, 2020*).

A schematic diagram is shown in Fig. S1 which represents the dynamic relations of this model.

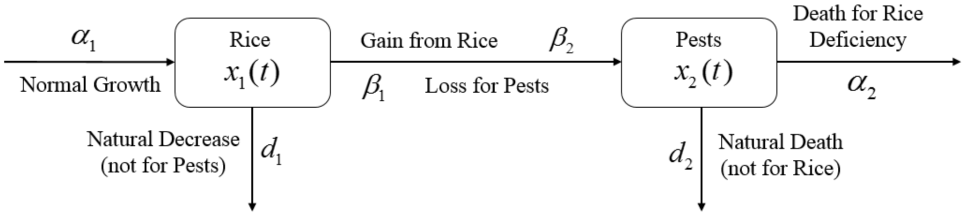

Figure S1 A schematic diagram of the rice-pest system (S4) disclosing the interrelationship between rice and corresponding rice pests along with the impact of adverse environment.

Supplemental Information 1.2 - Positivity Analysis

In the following, we intend to show that the rice-pest system (S4) is bounded in a positive region and the dynamic species of the system (S4) consist of non-negative values at any time (*Dym, 2004*).

Theorem 1.1. *The rice-pest system (S4) is bounded in a positive region* $\Psi = \{(x_1(t), x_2(t)) \in \mathbb{R}_+^2\}$.

Proof Consider $N(t) = x_1(t) + x_2(t)$ and $\mu \in \mathbb{R}^+$, then we can write

$$\frac{dN(t)}{dt} + \mu N(t) = \frac{d\,x_1(t)}{dt} + \frac{dx_2(t)}{dt} + \mu x_1(t) + \mu x_2(t)$$

$$\Rightarrow \frac{dN(t)}{dt} + \mu N(t) = (\alpha_1 - \beta_1 x_2(t))x_1(t) - d_1 x_1^{\,2}(t) - (\alpha_2 - \beta_2 x_1(t))x_2(t) - d_2 x_2^{\,2}(t) + \mu x_1(t) + \mu x_2(t) \tag{S5}$$

Since the consumption of pests and the losses of rice are approximately equal at the equilibrium point i.e., $\beta_1 \approx \beta_2$, Eq. (S5) takes the following form

$$\frac{dN(t)}{dt} + \mu N(t) = (\alpha_1 + \mu)x_1(t) - d_1 x_1^{\,2}(t) + (\mu - \alpha_2)x_2(t) - d_2 x_2^{\,2}(t)$$

$$\Rightarrow \frac{dN(t)}{dt} + \mu N(t) \le \frac{1}{4d_1}(\alpha_1 + \mu)^2 + \frac{1}{4d_2}(\mu - \alpha_2)^2 \tag{S6}$$

Applying the principle of differential inequalities in Eq. (S6), we get

$$0 \le N(t)\left(x_1(t), x_2(t)\right) \le \frac{\xi}{\mu}\left(1 - e^{-\mu t}\right) + (x_{10} + x_{20})e^{-\mu t}, \text{ where } \xi = \frac{1}{4d_1}(\alpha_1 + \mu)^2 + \frac{1}{4d_2}(\mu - \alpha_2)^2$$

Taking the limit as $t \to \infty$ we obtain $0 \le N(t) \le \frac{\xi}{\mu}$, hence the region of attraction for the system can be formulated as $\Psi = \left\{\left(x_1(t), x_2(t)\right) \in \mathbb{R}_+^2 : N(t) = x_1(t) + x_2(t), 0 \le N(t) \le \frac{\xi}{\mu}\right\}$ with $\xi, \mu \in \mathbb{R}^+$. This indicates that the system is positively bounded in Ψ.

Theorem 1.2. *Each species of the model (S4) contains a non-negative real value for all* $t \ge 0$.
Proof To estimate the solution, consider the first equation of the model (S4) given as

$$x_1'(t) = (\alpha_1 - \beta_1 x_2(t))x_1(t) - d_1 x_1^2(t) \qquad \text{where } \frac{dx_1(t)}{dt} = x_1'(t) \tag{S7}$$

According to the condition of positivity, Eq. (S7) takes the following form

$$x_1'(t) \le \alpha_1 x_1(t) - d_1 x_1^2(t) \;\Rightarrow \frac{1}{x_1(t)}\frac{dx_1(t)}{dt} \le \alpha_1 - d_1 x_1(t) \tag{S8}$$

After solving Eq. (S8), the solution of $x_1(t)$ becomes $x_1(t) \le \dfrac{\alpha_1 x_{10}}{(\alpha_1 - d_1 x_{10})e^{-\alpha_1 t} + d_1 x_{10}}$. Therefore, $\lim_{t\to\infty} x(t) \le \dfrac{\alpha_1}{d_1}$ i.e., $\dfrac{\alpha_1}{d_1}$ is the upper limit of $x_1(t)$ which implies that $x_{10} \le x_1(t) \le \dfrac{\alpha_1}{d_1}$ i.e., $x_1(t)$ is non-negatively bounded. Similarly, it can be shown that $x_{20} \le x_2(t) \le \dfrac{\alpha_2}{d_2}$. Hence the species of the system are positively bounded.

Supplemental Information 1.3 - Equilibrium Points

To conduct the stability analysis of the rice-pest system (S4), we first obtain the equilibria of the system, considering three different situations (*Dym, 2004*):

(i). The *trivial* or *pre-cultivation* case refers to the situation prior to cultivation. No plants and pests exist yet. Therefore, the pre-cultivating equilibrium point is $(x_{10}, x_{20}) = (0,\, 0)$.

(ii). The *pest-free* case refers to the pre-existing state of rice pests, indicating that there are rice plants, but no pests. Here, the pest-free equilibrium point is $(\bar{x}_1,\, 0) = \left(\dfrac{\alpha_1}{d_1},\, 0\right)$.

(iii). A *co-existence* or *farming* situation refers to a competitive case where both rice and pests coexist. The co-existing equilibrium point of the rice-pest system (S4) is

$$(\hat{x}_1,\, \hat{x}_2) = \left(\frac{\alpha_1 d_2 + \alpha_2 \beta_1}{d_1 d_2 + \beta_1 \beta_2},\, \frac{\alpha_1 \beta_2 - \alpha_2 d_1}{d_1 d_2 + \beta_1 \beta_2}\right).$$

Supplemental Information 1.4 - Stability Analysis

To illustrate the nature of the equilibrium points of the model (S4), a stability analysis has been conducted. In this case, let's consider system (S4) in the following vectorial form (*Dym, 2004*)

$$\begin{cases} x'(t) = f(\bar{x}, t) \\ f = (f_1, f_2) \end{cases} \tag{S9}$$

where $f_1(x(t),t) = (\alpha_1 - \beta_1 x_2(t))x_1(t) - d_1 x_1^2(t)$ and $f_2(x(t),t) = (\beta_2 x_1(t) - \alpha_2)x_2(t) - d_2 x_2^2(t)$

After evaluating the vector field, the Jacobian matrix is obtained as the following

$$J = \begin{bmatrix} \alpha_1 - \beta_1 x_2(t) - 2d_1 x_1(t) & -\beta_1 x_1(t) \\ \beta_2 x_2(t) & -\alpha_2 + \beta_2 x_1(t) - 2d_2 x_2(t) \end{bmatrix} \tag{S10}$$

Theorem 1.3. *System (S4) approaches a saddle-node at the pre-cultivation equilibrium.*
Proof To prove the theorem, evaluate the Jacobian matrix (S10) at (x_{10}, x_{20}) which becomes of the following form

$$J_{(x_{10},x_{20})} = \begin{bmatrix} \alpha_1 & 0 \\ 0 & -\alpha_2 \end{bmatrix} \leftrightarrow \det\left(J_{(x_{10},x_{20})} - I\lambda\right) = \begin{vmatrix} \alpha_1 - \lambda & 0 \\ 0 & -\alpha_2 - \lambda \end{vmatrix} \tag{S11}$$

The characteristic equation of Eq. (S11) is

$$(\alpha_1 - \lambda)(-\alpha_2 - \lambda) = \lambda^2 - (\alpha_1 - \alpha_2)\lambda - \alpha_1\alpha_2 = 0 \tag{S12}$$

Hence the eigenvalues of Eq. (S12) are $\lambda_1 = \alpha_1$ and $\lambda_2 = -\alpha_2$. Since the eigenvalues are real and are opposite in sign, the *pre-cultivating equilibrium* represents a saddle point.

For an autonomous system, an equilibrium point is a saddle point if the characteristic equation consists of opposite signed (one positive and one negative) real eigenvalues (*Youssef & Raffoul, 2022*). A stable point exhibits a mean position between the stability and instability of a point. For a biological example in two dimensions, the dynamic species appear to have reached a stable equilibrium situation but, their trajectory bends to the other side instead.

Theorem 1.4. *The system described by Eq. (S4) at pest-free equilibrium approaches a saddle point.*

Proof To test the stability at *pest-free equilibrium*, evaluate the Jacobian matrix (S10) at $(\bar{x}_1, 0)$ through its characteristic equation by calculating $\det\left(J_{(\bar{x}_1,\,0)} - I\lambda\right) = 0$

$$\therefore\ J_{(\bar{x}_1,\,0)} = \begin{bmatrix} -\alpha_1 & -\dfrac{\beta_1\alpha_1}{d_1} \\ 0 & \dfrac{\alpha_1\beta_2}{d_1} - \alpha_2 \end{bmatrix} \longleftrightarrow \det\left(J_{(\bar{x}_1,\,0)} - I\lambda\right) = \begin{vmatrix} -\alpha_1 - \lambda & -\dfrac{\beta_1\alpha_1}{d_1} \\ 0 & \dfrac{\alpha_1\beta_2}{d_1} - \alpha_2 - \lambda \end{vmatrix} \tag{S13}$$

Therefore, the characteristic equation of Eq. (S13) is

$$\left(-\alpha_1 - \lambda\right)\left(\frac{\alpha_1\beta_2}{d_1} - \alpha_2 - \lambda\right) = \lambda^2 - \left(\alpha_1 + \alpha_2 - \frac{\alpha_1\beta_2}{d_1}\right)\lambda - \frac{\alpha_1{}^2\beta_2}{d_1} + \alpha_1\alpha_2 = 0 \tag{S14}$$

Similarly, the eigenvalues of Eq. (S14) become $\lambda_1 = -\alpha_1$, and $\lambda_2 = \dfrac{\alpha_1\beta_2 - \alpha_2 d_1}{d_1}$ with λ_2 being strictly positive since $\alpha_1\beta_2 > \alpha_2 d_1$ which indicates that the *pest-free equilibrium* represents a saddle point.

Theorem 1.5. *The rice-pest system (S4) approaches a spiral node at the co-existing equilibrium point.*

Proof Evaluating the Jacobian matrix (S10) at the co-existing equilibrium point, it takes the following form

$$J_{(\hat{x}_1,\,\hat{x}_2)} = \begin{bmatrix} -2\dfrac{\left(d_2\alpha_1 + \alpha_2\beta_1\right)d_1}{d_1d_2 + \beta_1\beta_2} + \dfrac{\left(\alpha_2 d_1 - \alpha_1\beta_2\right)\beta_1}{d_1d_2 + \beta_1\beta_2} + \alpha_1 & -\dfrac{\beta_1\left(d_2\alpha_1 + \alpha_2\beta_1\right)}{d_1d_2 + \beta_1\beta_2} \\ -\dfrac{\beta_2\left(\alpha_2 d_1 - \alpha_1\beta_2\right)}{d_1d_2 + \beta_1\beta_2} & \dfrac{\left(d_2\alpha_1 + \alpha_2\beta_1\right)\beta_2}{d_1d_2 + \beta_1\beta_2} + 2\dfrac{\left(\alpha_2 d_1 - \alpha_1\beta_2\right)d_2}{d_1d_2 + \beta_1\beta_2} - \alpha_2 \end{bmatrix} \tag{S15}$$

The characteristic equation of Eq. (S15) is again calculated via $\det\left(J_{(\hat{x}_1,\,\hat{x}_2)} - I\lambda\right) = 0$

$$\left|J_{(\hat{x}_1,\,\hat{x}_2)} - I\lambda\right| = \begin{vmatrix} -2\dfrac{\left(d_2\alpha_1 + \alpha_2\beta_1\right)d_1}{d_1d_2 + \beta_1\beta_2} + \dfrac{\left(\alpha_2 d_1 - \alpha_1\beta_2\right)\beta_1}{d_1d_2 + \beta_1\beta_2} + \alpha_1 - \lambda & -\dfrac{\beta_1\left(d_2\alpha_1 + \alpha_2\beta_1\right)}{d_1d_2 + \beta_1\beta_2} \\ -\dfrac{\beta_2\left(\alpha_2 d_1 - \alpha_1\beta_2\right)}{d_1d_2 + \beta_1\beta_2} & \dfrac{\left(d_2\alpha_1 + \alpha_2\beta_1\right)\beta_2}{d_1d_2 + \beta_1\beta_2} + 2\dfrac{\left(\alpha_2 d_1 - \alpha_1\beta_2\right)d_2}{d_1d_2 + \beta_1\beta_2} - \alpha_2 - \lambda \end{vmatrix} = 0$$

with the eigenvalues becoming

$$\lambda_1 = \frac{1}{2}\frac{1}{d_1d_2 + \beta_1\beta_2}\left[-w_1 + \sqrt{w_2 + 2w_3 - 4w_4}\right] \text{ and } \lambda_2 = -\frac{1}{2}\frac{1}{d_1d_2 + \beta_1\beta_2}\left[w_1 + \sqrt{w_2 + 2w_3 - 4w_4}\right]$$

with $w_1 = \alpha_1 d_2(\beta_2 + d_1) + d_1\alpha_2(\beta_1 - d_2)$, $w_2 = \alpha_1{}^2 d_2{}^2\left(\beta_2{}^2 + d_1{}^2\right) + \alpha_2{}^2 d_1{}^2\left(\beta_1{}^2 + d_2{}^2\right)$,
$w_3 = d_1d_2\left(\alpha_1\alpha_2 d_1(\beta_1 + d_2) + \alpha_2\beta_1(\alpha_2 d_1 + \alpha_1\beta_2) - \alpha_1\beta_2 d_2(\alpha_1 + \alpha_2)\right)$, and
$w_4 = \beta_1\beta_2\left(\alpha_1\alpha_2\beta_1\beta_2 + d_2\alpha_1{}^2\beta_2 - d_1\alpha_2{}^2\beta_1\right)$.

Both the eigenvalues are complex numbers which indicate that the *co-existing equilibrium* approaches a spiral node.

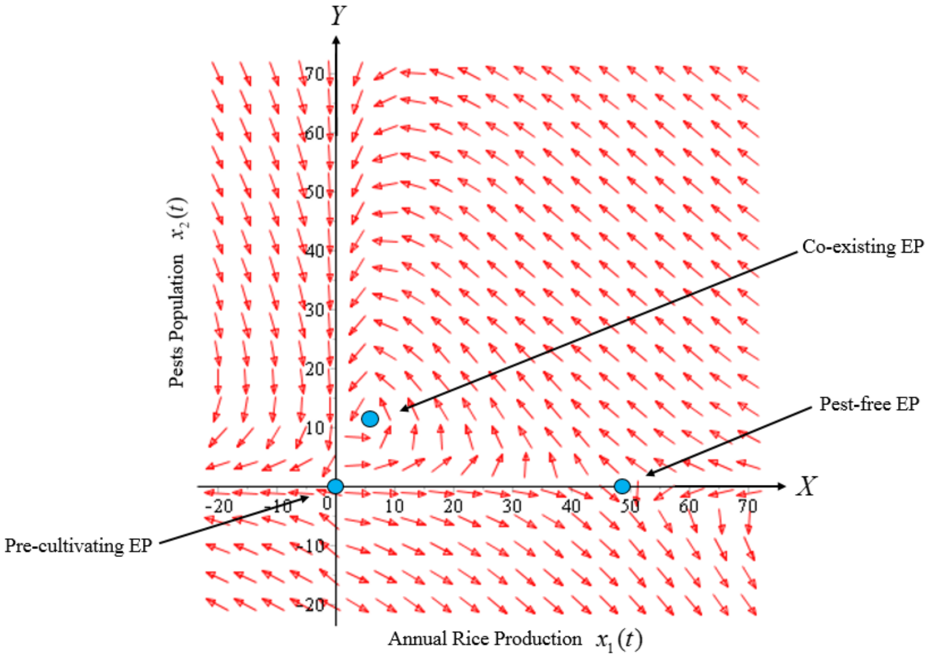

Figure S2 Phase portrait discloses the characteristics of three equilibrium points (EP) of the rice-pest system (S4). Here, the co-existing equilibrium point is a spiral node, and the others, pre-cultivating and pest-free equilibrium points, are saddle points.
