# Supplementary material for: Dynamic analysis and control of a rice-pest system under transcritical bifurcations
Source: PeerJ. 2023 Oct 10;11:e16083. doi: 10.7717/peerj.16083 (PMC10573297; doi:10.7717/peerj.16083)
Supplement: Supplemental Information 2 [file peerj-11-16083-s002.pdf]

Supplemental Information 2. Bifurcation analysis for the rice-pest system

In this section, we investigate the rice-pest system (S4) through transcritical bifurcation analysis (*Banerjee & Petrovskii, 2011*). For a transcritical bifurcation exists a non-destructible fixed point over the whole bifurcation parameter range, which, however, changes its stability characteristic for altered bifurcation parameters (*Banerjee & Petrovskii, 2011*). For the transcritical bifurcation analysis of the rice-pest system (S4), it is more convenient to use a dimensionless rice-pest system (S4) which reduces the number of parameters (*Banerjee & Petrovskii, 2011*). In this case, we introduce the dimensionless variables $x_1(t) = x^*.\hat{x}$ and $x_2(t) = y^*.\hat{y}$ and consider the dimensionless time $t = \tau^*\sigma$. Removing the symbols '*' and '^', the system (S4) becomes dimensionless given as

$$\begin{cases} \dfrac{dx}{d\tau} = x(\alpha - x) - xy \equiv f_1(x,y) \\ \dfrac{dy}{d\tau} = \beta xy - y - \gamma y^2 \equiv f_2(x,y) \end{cases} \tag{S16}$$

where $\sigma = \dfrac{1}{\alpha_2}$, $\alpha = \dfrac{\alpha_1}{\alpha_2}$, $\beta = \dfrac{\beta_2}{d_1}$, $\gamma = \dfrac{d_2}{\beta_1}$.

The biologically meaningful equilibria of the system (S16) are the non-negative solutions of $f_1(x,y) = 0$ and $f_2(x,y) = 0$. The rice (prey) isocline consists of the axis $x = 0$ and the straight line $y = \alpha - x$ and the pests (predator) isocline consists of the axis $y = 0$ and the line $x = \dfrac{1+\gamma y}{\beta}$ (*Banerjee & Petrovskii, 2011*). Correspondingly, the system (S16) is bounded by two equilibrium points, the trivial/pre-cultural equilibrium $E_0(x,y) = (0,0)$ and the pest-free equilibrium $E_1(x,y) = (\alpha,0)$. An interior equilibrium $E_*(x,y) = (x_*, y_*)$ with $(x_*, y_*) = \left(\dfrac{1+\gamma y_*}{\beta}, \alpha - x_*\right) = \left(\dfrac{\alpha\gamma+1}{\beta+\gamma}, \dfrac{\alpha\beta-1}{\beta+\gamma}\right)$ can be found at the intersection of the two isoclines. For the existence of E_*, the value of the parameters must follow the conditions $\alpha\gamma + 1 \ge 0$, $\beta + \gamma > 0$ and $\alpha\beta - 1 \ge 0$.

Theorem 2.1. *The system (S16) experiences transcritical bifurcation at the pest-free equilibrium point* $E_1(\alpha,0)$ *as the growth parameter* α *passes through the critical value* α^*.

Proof At the pest-free equilibrium point $E_1(\alpha,0)$, the associated Jacobian matrix of the system (S16) takes the form:

$$J_{E_1}(\alpha^*) = \begin{bmatrix} -\alpha & 0 \\ 0 & 0 \end{bmatrix} \tag{S17}$$

The set of eigenvalues of $J_{E_1}(\alpha^*)$ is $\lambda = \begin{bmatrix} -\alpha \\ 0 \end{bmatrix}$ i.e., one eigenvalue is zero and the other is negative since $\alpha > 0$. Therefore, to examine the nature of the system at E_1, we have applied *Sotomayor's theorem* (Perko, 2000). For this purpose, we consider the system (S16) as

$$f(x,y) = \begin{pmatrix} f_1(x,y) \\ f_2(x,y) \end{pmatrix} \tag{S18}$$

Let the eigenvectors corresponding to the zero eigenvalues of $J_{E_1}(\alpha^*)$ and $J_{E_1}{}^T(\alpha^*)$ be V and W, respectively, where $V = W = \begin{bmatrix} 1 \\ 0 \end{bmatrix}$. From Eq. (S18), we have $f_\alpha(E_1;\alpha^*) = \begin{bmatrix} \alpha \\ 0 \end{bmatrix}$, $Df_\alpha(E_1;\alpha^*) = \begin{bmatrix} \alpha & 0 \\ 0 & 0 \end{bmatrix}$ and $D^2 f(E_1;\alpha^*)(V,V) = \begin{bmatrix} -2 \\ 0 \end{bmatrix}$. Here, Df denotes the partial derivative of f with respect to x and y, and Df_α denotes the partial derivative of f with respect to the parameter α. Therefore,

$$W^T f_\alpha(E_1;\alpha^*) = \alpha \ne 0$$

$$W^T \left[Df_\alpha(E_1;\alpha^*) V \right] = \alpha \ne 0$$

$$W^T\left[D^2 f(E_1;\alpha^*)(V,V)\right]=-2\neq 0$$

Hence, there is a saddle-node bifurcation at the nonhyperbolic equilibrium point $E_1(\alpha,0)$ at the bifurcation value α. For $\alpha<0$, there is no equilibrium point. For $\alpha=0$, the $f_1(x,y)=-x^2$ is structurally unstable and the bifurcation value $\alpha=0$. Therefore, there is a transcritical bifurcation at the origin for $\alpha=0$. There are two equilibria at origin $(0,0)$ and $E_1(\alpha,0)$ (*Perko, 2000*).

Theorem 2.2. *The system (S16) experiences a transcritical bifurcation at the equilibrium point* $E_*(x_*,y_*)$ *as the growth parameter of the pest species population* β *passes through the critical value* β^*.

Proof At the equilibrium point $E_*(x_*,y_*)$, the associated Jacobian matrix of the system (S16) takes the form:

$$J_{E_*}(\beta^*)=\begin{bmatrix}-\dfrac{\alpha\gamma+1}{\beta+\gamma} & -\dfrac{\alpha\gamma+1}{\beta+\gamma}\\ \dfrac{\alpha\beta^2-\beta}{\beta+\gamma} & \dfrac{\gamma(1-\alpha\beta)}{\beta+\gamma}\end{bmatrix}. \tag{S19}$$

It can be shown that one eigenvalue of $J_{E_*}(\beta^*)$ is negative and the other one is zero for the condition $\alpha\beta=1$ (*Perko, 2000*). To investigate the nature of the system at E_*, we have applied *Sotomayor's theorem* (*Sen, Banerjee & Morozov, 2012*). Let the eigenvectors correspond to the zero eigenvalues of $J_{E_*}(\beta^*)$ and $J_{E_*}{}^T(\beta^*)$ be V and W, respectively, where $V=\begin{bmatrix}1\\-1\end{bmatrix}$ and $W=\begin{bmatrix}0\\1\end{bmatrix}$. From Eq. (S18), we have $f_\beta(E_*;\beta^*)=\begin{bmatrix}0\\x_*y_*\end{bmatrix}$, $Df_\beta(E_*;\beta^*)=\begin{bmatrix}0 & 0\\ y_* & x_*\end{bmatrix}$ and $D^2 f(E_*;\beta^*)(V,V)=\begin{bmatrix}0\\-2\beta-2\gamma\end{bmatrix}$. Therefore,

$$W^T f_\beta(E_*;\beta^*)=0$$

$$W^T\left[Df_\beta(E_*;\beta^*)V\right]=y_*-x_*\neq 0$$

$$W^T\left[D^2 f(E_*;\beta^*)(V,V)\right]=-2\beta-2\gamma\neq 0$$

Hence the system (S16) satisfies all the necessary conditions of *Sotomayor's theorem* and thus the system (S16) experiences a transcritical bifurcation at the co-existence equilibrium point E_* for the bifurcation parameter β (*Perko, 2000*).

Supplemental Information 2.1 – Finding values for dimensionless parameters

We have numerically investigated the dynamic behaviour of the system (S16) for the variation in the growth of pest populations (β). Let β_0 be the initial condition for the existence of E_*. The parameters must follow the conditions $\alpha\gamma+1\geq 0$, $\beta+\gamma>0$ and $\alpha\beta-1\geq 0$ and to estimate β_0, we consider $\alpha=1$ and $\gamma=0.001$ which satisfy all the conditions. Therefore, we get $\beta_0=1$ after calculating $\det\left(J_{E_*}(\beta^*)\right)=0$ (*Sen, Banerjee & Morozov, 2012*).

Supplemental Information 2.2 – A supportive figure

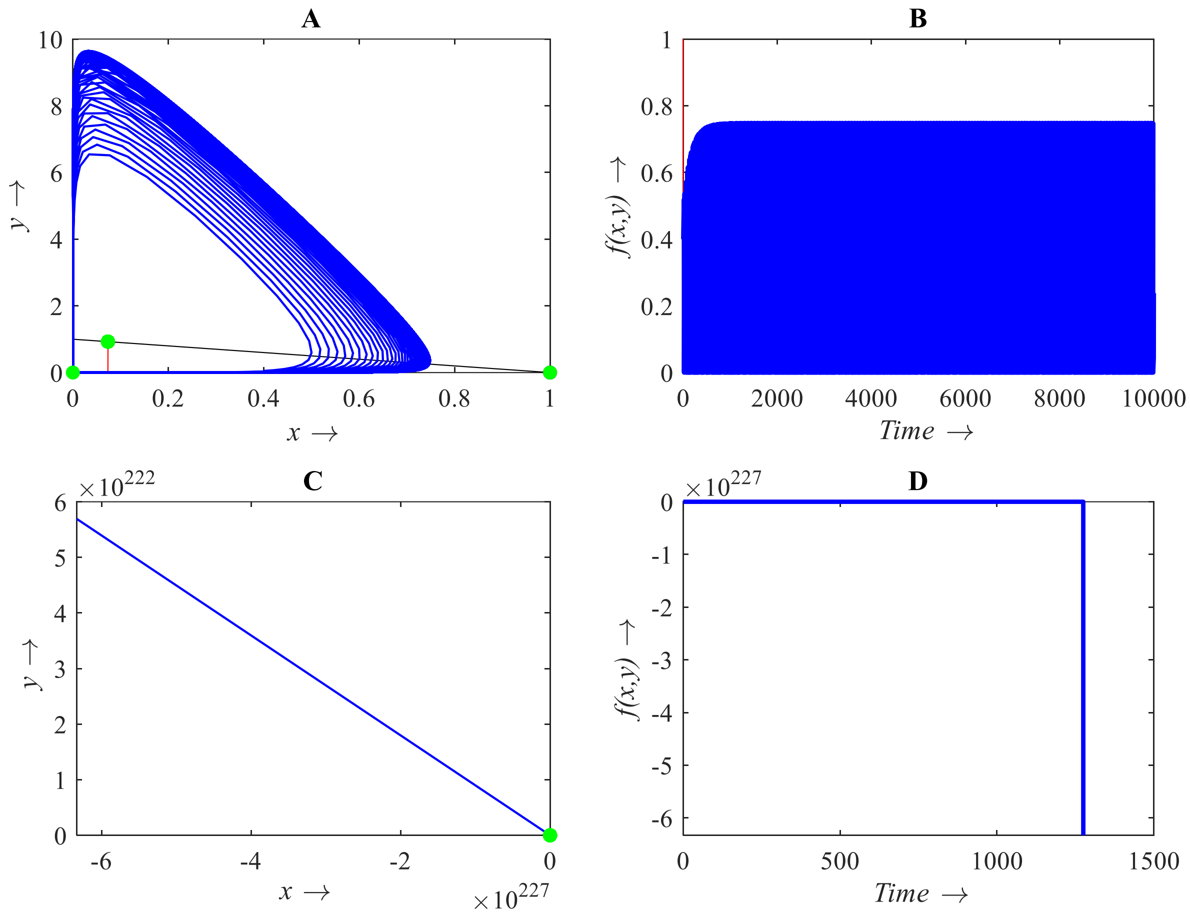

Figure S3 (A) Phase plane of the rice-pest system (S16) for $\beta = 13.6$, (B) time series analysis of (A), (C) phase plane of the system (S16) for $\beta = 13.7$, and (D) time series analysis of (C). The system experiences a steady-state limit cycle for $\beta = 13.6$, and approaches $E_1(0,0)$ for $\beta = 13.7$ meaning that the system exticts over a long time.
