# Supplementary material for: Dynamic analysis and control of a rice-pest system under transcritical bifurcations
Source: PeerJ. 2023 Oct 10;11:e16083. doi: 10.7717/peerj.16083 (PMC10573297; doi:10.7717/peerj.16083)
Supplement: Supplemental Information 3 [file peerj-11-16083-s003.pdf]

Supplemental Information 3. Data Analysis

All the parameters are secondary (derived/estimated or collected from other sources), two of which are estimated. For parametric estimation, we first studied deeply the rice-pest system. Then we conducted statistical analysis for parametric estimation after collecting and observing the corresponding data collected from different sources (*FAO, 2021*; *Tsuruishi, 2003*; *Vargas & Nishida, 1980*). The estimation of parameters used in this study is described below.

Estimation of α_1

Table A1 Annual data of the production of rice (*FAO, 2021*)

Data\Years	2018	2013	2008	2003	1998	1993	1988	1983	1978	1973	1968	1963
Harvested area (MH)	167.12	165.12	160.08	148.45	151.68	146.45	146.40	142.83	143.50	136.57	129.26	120.15
Production (MMT)	782.00	742.50	687.05	586.93	578.81	529.60	487.46	448.02	385.21	334.93	288.62	247.12
Production rate (metric tons/ha)	4.679* (α_1)	4.497	4.292	3.954	3.816	3.616	3.330	3.137	2.684	2.452	2.286	2.057

Here, MH presents Millions of hectares, and MMT means Millions of metric tons.

Therefore, the production rate of rice is α_1 = 4.679 metric tons/ hectare

Estimation of α_2

Several pest species may have in paddy fields, but we don't know how many species and which species because the number of pest species varies depending on several factors such as paddy species, locations, and availability of water (*FAO, 2021*). To make it simple, we assumed that minimum two pest species must be found in paddy fields. Therefore, two almost common pest species have been considered, presented in Table A2 and Table A3, to calculate the average mortality rate of the pest population.

Table A2 Life table for *Heliothis zea* in sweet corn 10 October 1976 – 20 November 1976 (*Vargas & Nishida, 1980*)

Age interval	Number alive at the beginning	Mortality rate
Egg	1000 (initial number)	Mortality rate = (initial number-final living number) / initial number<br>$M_1 = \frac{1000-52}{1000} = 0.948 = 94.8\%$
Larva	71	
Pupa	70	
Adult	52 (final living number)	

Table A3 Cohort life table from a hypothetical caddisfly population (*Tsuruishi, 2003*).

Age interval	Number alive at the beginning	Mortality rate
Egg	44,000 (initial number)	Mortality rate = (initial number-final living number) / initial number<br>$M_2 = \frac{44000-2187}{44000} = 0.9503 = 95.03\%$
Larva	2300	
Pupa	2250	
Adult	2187 (final living number)	

The average mortality rate is $M = \frac{M_1 + M_2}{2} = \frac{94.8\% + 95.03\%}{2} = 94.915\%$

$\therefore \alpha_2 = 94.915\%$

Estimation of β_1 and β_2

Annual losing rate of rice by pests is about 37% =37/100= 0.37 (*Peshin & Dhawan, 2009*; *FAO, 2003*; *IRRI, 2020*). Therefore β_1 = 37% or 0.37.

Since the predation rate of predators is approximately equal to the loss rate of prey population in co-existing equilibrium (*Peshin & Dhawan, 2009*; *FAO, 2003*; *IRRI, 2020*), therefore $\beta_2 = \beta_1$ = 37% or 0.37.

Please note that since the predation of the predator population is the loss of the prey population according to the law of conservation of energy in biology (*Dym, 2004*), the consumption of rice by pests is the loss of rice production due to pest infestations. Some energy losses may exist there which are not considered significant. Therefore, the value of β_1 and β_2 are the same, although their meanings are different. There have some natural causes that decrease rice production, such as floods, drought, and climate change, we presented this loss due to natural causes by d_1.

Estimation of d_1 and d_2

The natural death rate of paddy plants (without pests) is approximately 10% (on average) (*FAO, 2021*). Therefore d_1 =10% or 0.10. The death rate of pests due to natural causes or chemical control = (1 – normal mortality rate of pests) where 1 is the total probability index. Therefore, d_2 = (1 - α_2) = (1 - 0.94915) = 0.05085 = 5.085%.

Estimation of x_{10}

The term "x_{10}" presents the initial annual production of rice in metric tons per hectare arena (Mt/h) and it was 4.679 Mt/h in 2018, the same value as α_1 (*FAO, 2021*). The calculation is presented in Table A1.

Estimation of x_{20}

The term "x_{20}" presents the initial growth rate of pests. Since the death rate of pests is found 94.915%, the value of α_2 (*Vargas & Nishida, 1980*; *Tsuruishi, 2003*). Therefore, the initial growth rate of pests, $x_{20} = 1 - \alpha_2 =$ 0.05085.

The parameters with numerical values are displayed in Table 1. The parameters are also verified by comparing the results with other research (*Inao et al., 2008*; *Marinelli, 2005*; *Peshin & Dhawan, 2009*; *IPM, 2021*; *Oerke et al., 1994*; *Milligan, et al., 2016*), employing numerical simulations as described in more detail in Results.
