# Supplementary material for: Dynamic analysis and control of a rice-pest system under transcritical bifurcations
Source: PeerJ. 2023 Oct 10;11:e16083. doi: 10.7717/peerj.16083 (PMC10573297; doi:10.7717/peerj.16083)
Supplement: Supplemental Information 4 [file peerj-11-16083-s004.pdf]

Supplemental Information 4: MATLAB Scripts

****************************** **Rice-pest mode (S4)** ******************************

********************************* **Figure 4** ***********************************

```
clc
clear all
ts=[0:0.01:12]; % time
x0=[4.679 0.05085]; % initial conditions/values of x1 and x2
op1 = odeset('OutputFcn',@odephas2);
options = [op1];
T=[0:12]; % time
[T,X]=ode45(@sajibcon, ts, x0,options);

%%
figure;

subplot(1,2,1)
 plot (T,X (:,1),'-r',T,X (:,2),'-g','LineWidth',2);
 xlabel('Time (Months)'), ylabel('Growth of Rice Pests');
 legend('Rice, x_1(1)','Pests, x_2(t)');
 grid off;

subplot(1,2,2)
 ts=[0:0.01:12];
 x0=[4.679 0.05085];
 op1 = odeset('OutputFcn',@odephas2);
 options = [op1];
 T=[0:12];
 [T,X]=ode45(@sajib2, ts, x0,options);
 xlabel('x_1(t)'), ylabel('x_2(t)');
 grid off;
```

```
function dx=sajibcon(ts,x)

a1=4.679; % alpha_1
a2=0.94915; % alpha_2
b1=0.37; % beta_1
b2=0.37; % beta_2
d1=0.10; % d_1
d2=0.05085; % d_2

dx=zeros (2,1);
dx(1)=(a1-b1*x(2))*x(1)-d1*x(1)^2; % equations of rice-pest model (S4)
dx(2)= -(a2-b2*x(1))*x(2)-d2*x(2)^2;
end
```

********************************** **Figure 5** **********************************

```
clc
clear all
ts=[0:0.001:12];
x0=[4.679 0.05085];
T=[0:12];

%%
figure;

subplot(1,2,1)
```

```
 [T,X]=ode45(@sajib4, ts, x0);
   plot(T,X(:, 1),'r','LineWidth',2);grid off;hold on
   xlabel('Time (Months)'), ylabel('Growth of Rice, x_1(t)');
 [T,X]=ode45(@sajib3, ts, x0);
   plot(T,X(:, 1),'g','LineWidth',2);grid off;hold on
 [T,X]=ode45(@sajib2, ts, x0);
   plot(T,X(:, 1),'b','LineWidth',2);grid off;hold on
 legend('\beta_2=0.15','\beta_2=0.25','\beta_2=0.37');hold on

subplot(1,2,2)
 [T,X]=ode45(@sajib4, ts, x0);
   plot(T,X(:, 2),'r','LineWidth',2);grid off;hold on
   xlabel('Time (Months)'), ylabel('Growth of Pests, x_2(t)');
 [T,X]=ode45(@sajib3, ts, x0);
   plot(T,X(:, 2),'g','LineWidth',2);grid on;hold on
 T,X]=ode45(@sajib2, ts, x0);
   plot(T,X(:, 2),'b','LineWidth',2);grid off;hold on
 legend('\beta_2=0.15','\beta_2=0.25','\beta_2=0.37');hold on

---------------------------------------------------------------------------
function dx=sajib2(ts,x)

a1=4.679;
a2=0.94915;
b1=0.37;
b2=0.37; %
d1=0.10;
d2=0.05085;

dx=zeros (2,1);
dx(1)=(a1-b1*x(2))*x(1)-d1*x(1)^2;
dx(2)= -(a2-b2*x(1))*x(2)-d2*x(2)^2;
end
---------------------------------------------------------------------------
function dx=sajib3(ts,x)

a1=4.679;
a2=0.94915;
b1=0.37;
b2=0.25; %
d1=0.10;
d2=0.05085;

dx=zeros (2,1);
dx(1)=(a1-b1*x(2))*x(1)-d1*x(1)^2;
dx(2)= -(a2-b2*x(1))*x(2)-d2*x(2)^2;
end
---------------------------------------------------------------------------
function dx=sajib4(ts,x)

a1=4.679;
a2=0.94915;
b1=0.37;
b2=0.15; %
d1=0.10;
d2=0.05085;

dx=zeros (2,1);
dx(1)=(a1-b1*x(2))*x(1)-d1*x(1)^2;
dx(2)= -(a2-b2*x(1))*x(2)-d2*x(2)^2;
end
---------------------------------------------------------------------------
```

**************************** Rice-pest-control model (7) ****************************

*********************************** Figure 6 *************************************

```
clc
clear all
ts=[0:0.001:12];
x0=[4.679 0.05085];
T=[0:12];

%%
figure;
subplot(1,3,1)
 [T,X]=ode45(@sajibcon, ts, x0); %sajibcon is provided in Figure 4 section
   plot(T,X(:, 1),'r','LineWidth',2);grid off;hold on
   xlabel('Time (Months)'), ylabel('Growth of Rice, x_1(t)');
 [T,X]=ode45(@sajibcon11, ts, x0);
   plot(T,X(:, 1),'--b','LineWidth',2);grid off;hold on
 legend('without control','with control u_1');hold on

subplot(1,3,2)
 [T,X]=ode45(@sajibcon, ts, x0); %sajibcon is provided in Figure 4 section
   plot(T,X(:, 2),'g','LineWidth',2);grid off;hold on
   xlabel('Time (Months)'), ylabel('Growth of Pests, x_2(t)');
 [T,X]=ode45(@sajibcon11, ts, x0);
   plot(T,X(:,2),'--b','LineWidth',2);grid off;hold on
 legend('without control','with control u_1');hold on

subplot(1,3,3)
    ts=[0:0.01:12];
    x0=[4.679 0.05085];
    op1 = odeset('OutputFcn',@odephas2);
    options = [op1];
    T=[0:15];
    [T,X]=ode45(@sajibcon11, ts, x0,options);
    xlabel('x_1(t)'), ylabel('x_2(t)');
    grid off;
```

```
function dx=sajibcon11(ts,x)

a1=4.679; % alpha_1
a2=0.94915; % alpha_2
b1=0.37; % beta_1
b2=0.37; % beta_2
d1=0.10; % d_1
d2=0.05085; % d_2

u1=0.2; % control variable
u2=0; % control variable

dx=zeros (2,1);
dx(1)=(a1-b1*x(2))*x(1)-d1*x(1)^2+u1*x(1);%Eqs. of rice-pest-control model (7)
dx(2)= -(a2-b2*x(1))*x(2)-d2*x(2)^2-(u1+u2)*x(1)*x(2);
end
```

********************************* Figure 7 *************************************

```
clc
clear all
ts=[0:0.001:12];
```

```
x0=[4.679 0.05085];
T=[0:12];

%%
figure;

subplot(1,3,1)
 [T,X]=ode45(@sajibcon, ts, x0); %sajibcon is provided in Figure 4 section
   plot(T,X(:, 1),'r','LineWidth',2);grid off;hold on
   xlabel('Time (Months)'), ylabel('Growth of Rice, x_1(t)');

 [T,X]=ode45(@sajibcon12, ts, x0);
   plot(T,X(:, 1),'--b','LineWidth',2);grid off;hold on
 legend('without control','with control u_2');hold on

subplot(1,3,2)
 [T,X]=ode45(@sajibcon, ts, x0); %sajibcon is provided in Figure 4 section
   plot(T,X(:, 2),'g','LineWidth',2);grid off;hold on
   xlabel('Time (Months)'), ylabel('Growth of Pests, x_2(t)');
 [T,X]=ode45(@sajibcon12, ts, x0);
   plot(T,X(:,2),'--b','LineWidth',2);grid off;hold on
 legend('without control','with control u_2');hold on

subplot(1,3,3)
    ts=[0:0.01:12];
    x0=[4.679 0.05085];
    op1 = odeset('OutputFcn',@odephas2);
    options = [op1];
    T=[0:12];
    [T,X]=ode45(@sajibcon12, ts, x0,options);
    xlabel('x_1(t)'), ylabel('x_2(t)');
    grid off;

---------------------------------------------------------------------------
function dx=sajibcon12(ts,x)

a1=4.679;
a2=0.94915;
b1=0.37;
b2=0.37;
d1=0.10;
d2=0.05085;

u1=0;
u2=0.26;

dx=zeros (2,1);
dx(1)=(a1-b1*x(2))*x(1)-d1*x(1)^2+u1*x(1);
dx(2)= -(a2-b2*x(1))*x(2)-d2*x(2)^2-u1*x(1)*x(2)-u2*x(1)*x(2);
end
---------------------------------------------------------------------------
```

******************************** **Figure 8** ***********************************

```
clc
clear all
ts=[0:0.001:12];
x0=[4.679 0.05085];
T=[0:12];

%% figure;
```

```
subplot(1,3,1)
 [T,X]=ode45(@sajibcon, ts, x0); %sajibcon is provided in Figure 4 section
   plot(T,X(:, 1),'r','LineWidth',2);grid off;hold on
   xlabel('Time (Months)'), ylabel('Growth of Rice, x_1(t)');
 [T,X]=ode45(@sajibcon13, ts, x0);
   plot(T,X(:, 1),'--b','LineWidth',2);grid off;hold on
 legend('without control','with control u_1 & u_2');hold on

subplot(1,3,2)
 [T,X]=ode45(@sajibcon, ts, x0); %sajibcon is provided in Figure 4 section
   plot(T,X(:, 2),'g','LineWidth',2);grid off;hold on
   xlabel('Time (Months)'), ylabel('Growth of Pests, x_2(t)');
 [T,X]=ode45(@sajibcon13, ts, x0);
   plot(T,X(:,2),'--b','LineWidth',2);grid off;hold on
 legend('without control','with control u_1 & u_2');hold on

subplot(1,3,3)
    ts=[0:0.01:12];
    x0=[4.679 0.05085];
    op1 = odeset('OutputFcn',@odephas2);
    options = [op1];
    T=[0:12];
     [T,X]=ode45(@sajibcon13, ts, x0,options);
    xlabel('x_1(t)'), ylabel('x_2(t)');
    grid off;
---------------------------------------------------------------------------
function dx=sajibcon13(ts,x)

a1=4.679;
a2=0.94915;
b1=0.37;
b2=0.37;
d1=0.10;
d2=0.05085;

u1=0.1;
u2=0.2;

dx=zeros (2,1);
dx(1)=(a1-b1*x(2))*x(1)-d1*x(1)^2+u1*x(1);
dx(2)= -(a2-b2*x(1))*x(2)-d2*x(2)^2-u1*x(1)*x(2)-u2*x(1)*x(2);
---------------------------------------------------------------------------
```

********************************* **Figure 9** **********************************

```
clc
clear all
ts=[0:0.001:12];
x0=[4.679 0.05085];
T=[0:12];

%%
figure;

subplot(1,2,1)
 [T,X]=ode45(@sajibcon, ts, x0); %sajibcon is provided in Figure 4
   plot(T,X(:, 1),'r','LineWidth',2);grid off;hold on
   xlabel('Time (Months)'), ylabel('Growth of Rice, x_1(t)');
 [T,X]=ode45(@sajibcon11, ts, x0); %sajibcon11 is provided in Figure 6
   plot(T,X(:,1),'m--','LineWidth',2);grid off;hold on
 [T,X]=ode45(@sajibcon12, ts, x0); %sajibcon12 is provided in Figure 7
   plot(T,X(:, 1),'g-.','LineWidth',2);grid off;hold on
```

```
 [T,X]=ode45(@sajibcon13, ts, x0); %sajibcon13 is provided in Figure 8
   plot(T,X(:, 1),'b:','LineWidth',2);grid off;hold on
 legend('without control','with control u_1','with control u_2','with
control u_1 & u_2');hold on

subplot(1,2,2)
 [T,X]=ode45(@sajibcon, ts, x0); %sajibcon is provided in Figure 4
   plot(T,X(:, 2),'r','LineWidth',2);grid off;hold on
   xlabel('Time (Months)'), ylabel('Growth of Pests, x_2(t)');
 [T,X]=ode45(@sajibcon11, ts, x0); %sajibcon11 is provided in Figure 6
   plot(T,X(:,2),'m--','LineWidth',2);grid off;hold on
 [T,X]=ode45(@sajibcon12, ts, x0); %sajibcon12 is provided in Figure 7
   plot(T,X(:, 2),'g-.','LineWidth',2);grid off;hold on
 [T,X]=ode45(@sajibcon13, ts, x0); %sajibcon13 is provided in Figure 8
   plot(T,X(:,2),'b:','LineWidth',2);grid off;hold on
 legend('without control','with control u_1','with control u_2','with
control u_1 & u_2');hold on
------------------------------------------------------------------------
```

******************************* Bifurcation analysis ********************************

********************************* Figure 10 *************************************

```
clear all
format long
set(0,'DefaultAxesFontSize',20);

%%
figure;

subplot(1,3,1)
  a=1; % alpha (dimensionless rice-pest model (S16))
  b=0.8; % beta (dimensionless rice-pest model (S16))
  g=0.001; % gamma (dimensionless rice-pest model (S16))

  X1=0:0.01:1;
  Y1=(a-X1); % y* value of the interior equilibrium point E*
  Y2=0:0.1:1;
  X2(1:length(Y2))=(1+g*Y2)/b; % x* value of the interior equilibrium point E*
  plot(X1,Y1,'k-');
  hold on;
  plot(X2,Y2,'r-');
  hold on
  xlabel('x \rightarrow');
  ylabel('y \rightarrow');

subplot(1,3,2)
  a=1;
  b=1; % beta
  g=0.001;

  X1=0:0.01:1;
  Y1=(a-X1);
  Y2=0:0.1:1;
  X2(1:length(Y2))=(1+g*Y2)/b;
  plot(X1,Y1,'k-');
  hold on;
  plot(X2,Y2,'r-');
  hold on
  xlabel('x \rightarrow');
  ylabel('y \rightarrow');
```

```
subplot(1,3,3)
  a=1;
  b=2; % beta
  g=0.001;

  X1=0:0.01:1;
  Y1=(a-X1);
  Y2=0:0.1:1;
  X2(1:length(Y2))=(1+g*Y2)/b;
  plot(X1,Y1,'k-');
  hold on;
  plot(X2,Y2,'r-');
  xlabel('x \rightarrow');
  ylabel('y \rightarrow');
  grid off;
```

****************************** General code for Figure 11 ************************

```
clear all
set(0,'DefaultAxesFontSize',20);

  alpha=1;
  beta=11.1; % the system is unstable when the beta is less than or equal to 1
             % the system is stable and doesn't show a limit cycle when beta = (1, 10.9]
             % the system is a stable limit cycle when beta = 11.0
             % the system shows a limit cycle that is a steady state when beta = (11.1, 13.6]
             % the system shows only a limit cycle when beta = 12.66
             % the system is unstable when beta > 13.6

  g=0.001; % gamma
  M=100000; % iteration number
  x=zeros(1,M+1);
  y=zeros(1,M+1);
  t=zeros(1,M+1);
  x(1)= 0.4; % initial value of x for the dimensionless model (S16)
  y(1)=0.05; % initial value of y for the dimensionless model (S16)
  t(1)=0; % initial time
  dt=.1; % time increment

  for j=1:M
    t(j+1)=t(j)+dt;
    x(j+1)=x(j)+dt*(alpha*x(j)-x(j)^2-x(j)*y(j)); % model (S16)
    y(j+1)=y(j)+dt*(beta*x(j)*y(j)-y(j)-g*y(j)^2); % model (S16)
  end

plot(x,y,'k','MarkerSize',10);
xlabel('x \rightarrow');
ylabel('y \rightarrow');

%plot(t,x,'b-','LineWidth',2);
```

********************************** **Figure 11** **********************************

```
clear all
set(0,'DefaultAxesFontSize',20);
  alpha=1;
  g=0.001;

  M=100000;
  x=zeros(1,M+1);
  y=zeros(1,M+1);
  t=zeros(1,M+1);
  x(1)= 0.4;
  y(1)=0.05;
```

```
  t(1)=0;
  dt=.1;

%% figure;

subplot(2,3,1)
  beta=2; %
  X1=0:0.01:1;
  Y1=(alpha-X1);
  Y2=0:0.1:1;
  X2(1:length(Y2))=(1+g*Y2)/beta;
  plot(X1,Y1,'k-');
  hold on;
  plot(X2,Y2,'r-');

  for j=1:M
    t(j+1)=t(j)+dt;
    x(j+1)=x(j)+dt*(alpha*x(j)-x(j)^2-x(j)*y(j));
    y(j+1)=y(j)+dt*(beta*x(j)*y(j)-y(j)-g*y(j)^2);
  end

plot(x,y,'b','MarkerSize',10, 'LineWidth',1);
xlabel('x \rightarrow');
ylabel('y \rightarrow');
X1(1:length(Y1))=(1+g*Y1)/beta;
Y1=(alpha-X1);
plot(0,0,'g.',alpha,0,'k.',X1,Y1,'g.',1,0,'g.','MarkerSize',20);


subplot(2,3,2)
  beta=10.9; %
  X1=0:0.01:1;
  Y1=(alpha-X1);
  Y2=0:0.1:1;
  X2(1:length(Y2))=(1+g*Y2)/beta;
  plot(X1,Y1,'k-');
  hold on;
  plot(X2,Y2,'r-');

  for j=1:M
    t(j+1)=t(j)+dt;
    x(j+1)=x(j)+dt*(alpha*x(j)-x(j)^2-x(j)*y(j));
    y(j+1)=y(j)+dt*(beta*x(j)*y(j)-y(j)-g*y(j)^2);
  end

plot(x,y,'b','MarkerSize',10, 'LineWidth',1);
xlabel('x \rightarrow');
ylabel('y \rightarrow');
X1(1:length(Y1))=(1+g*Y1)/beta;
Y1=(alpha-X1);
plot(0,0,'g.',alpha,0,'k.',X1,Y1,'g.',1,0,'g.','MarkerSize',20);


subplot(2,3,3)
  beta=11.1; %
  X1=0:0.01:1;
  Y1=(alpha-X1);
  Y2=0:0.1:1;
  X2(1:length(Y2))=(1+g*Y2)/beta;
  plot(X1,Y1,'k-');
  hold on;
```

```
  plot(X2,Y2,'r-');

  for j=1:M
    t(j+1)=t(j)+dt;
    x(j+1)=x(j)+dt*(alpha*x(j)-x(j)^2-x(j)*y(j));
    y(j+1)=y(j)+dt*(beta*x(j)*y(j)-y(j)-g*y(j)^2);
  end

plot(x,y,'b','MarkerSize',10, 'LineWidth',1);
xlabel('x \rightarrow');
ylabel('y \rightarrow');
X1(1:length(Y1))=(1+g*Y1)/beta;
Y1=(alpha-X1);
plot(0,0,'g.',alpha,0,'k.',X1,Y1,'g.',1,0,'g.','MarkerSize',20);


subplot(2,3,4)
  beta=12; %
  X1=0:0.01:1;
  Y1=(alpha-X1);
  Y2=0:0.1:1;
  X2(1:length(Y2))=(1+g*Y2)/beta;
  plot(X1,Y1,'k-');
  hold on;
  plot(X2,Y2,'r-');

  for j=1:M
    t(j+1)=t(j)+dt;
    x(j+1)=x(j)+dt*(alpha*x(j)-x(j)^2-x(j)*y(j));
    y(j+1)=y(j)+dt*(beta*x(j)*y(j)-y(j)-g*y(j)^2);
  end

plot(x,y,'b','MarkerSize',10, 'LineWidth',1);
xlabel('x \rightarrow');
ylabel('y \rightarrow');
X1(1:length(Y1))=(1+g*Y1)/beta;
Y1=(alpha-X1);
plot(0,0,'g.',alpha,0,'k.',X1,Y1,'g.',1,0,'g.','MarkerSize',20);


subplot(2,3,5)
  beta=12.66; %
  X1=0:0.01:1;
  Y1=(alpha-X1);
  Y2=0:0.1:1;
  X2(1:length(Y2))=(1+g*Y2)/beta;
  plot(X1,Y1,'k-');
  hold on;
  plot(X2,Y2,'r-');

  for j=1:M
    t(j+1)=t(j)+dt;
    x(j+1)=x(j)+dt*(alpha*x(j)-x(j)^2-x(j)*y(j));
    y(j+1)=y(j)+dt*(beta*x(j)*y(j)-y(j)-g*y(j)^2);
  end

plot(x,y,'b','MarkerSize',10, 'LineWidth',1);
xlabel('x \rightarrow');
ylabel('y \rightarrow');
X1(1:length(Y1))=(1+g*Y1)/beta;
Y1=(alpha-X1);
```

```
plot(0,0,'g.',alpha,0,'k.',X1,Y1,'g.',1,0,'g.','MarkerSize',20);


subplot(2,3,6)
  beta=13.6; %
  X1=0:0.01:1;
  Y1=(alpha-X1);
  Y2=0:0.1:1;
  X2(1:length(Y2))=(1+g*Y2)/beta;
  plot(X1,Y1,'k-');
  hold on;
  plot(X2,Y2,'r-');

  for j=1:M
    t(j+1)=t(j)+dt;
    x(j+1)=x(j)+dt*(alpha*x(j)-x(j)^2-x(j)*y(j));
    y(j+1)=y(j)+dt*(beta*x(j)*y(j)-y(j)-g*y(j)^2);
  end

plot(x,y,'b','MarkerSize',10, 'LineWidth',1);
xlabel('x \rightarrow');
ylabel('y \rightarrow');
X1(1:length(Y1))=(1+g*Y1)/beta;
Y1=(alpha-X1);
plot(0,0,'g.',alpha,0,'k.',X1,Y1,'g.',1,0,'g.','MarkerSize',20);
```

********************************** **Figure 12** ********************************

```
clear all
format long
set(0,'DefaultAxesFontSize',20)

for k=1:100
    b=1+(k-1)*0.01; % beta
dt=0.01; % time increment
M=500000; % time iteration

a=1;
b=2;
g=0.001;

X=zeros(1,M+1);
Y=zeros(1,M+1);

X(1)=0.4;
Y(1)=0.05;

      for j=1:M
            X(j+1)=X(j)+dt*(a*X(j)-X(j)^2-X(j)*Y(j));
            Y(j+1)=Y(j)+dt*(b*X(j)*Y(j)-Y(j)-g*Y(j)^2);
      end

XX(k)=max(X(end-5000:end));
YY(k)=min(X(end-5000:end));
k=k

end
```

```
beta=0:0.01:20;
x=(1*0.001+1)./(beta+0.001);
plot(beta,x);

plot(beta(1:101),x(1:101),'r-','LineWidth',2);
hold on
plot(beta(101:1110),x(101:1110),'b-','LineWidth',2);
plot(beta(1110:1370),x(1110:1370),'b-.','LineWidth',2);
plot(beta(1370:end),x(1370:end),'r-','LineWidth',2);

beta1=0:0.01:20;
x1(1:length(beta1))=1;
plot(beta1(1:101),x1(1:101),'r-','LineWidth',2);
plot(beta1(101:1110),x1(101:1110),'b-','LineWidth',2);
plot(beta1(1110:1370),x1(1110:1370),'b-.','LineWidth',2);
plot(beta1(1370:end),x1(1370:end),'r-','LineWidth',2);

axis([0 20 0 1.2]);
xlabel('\beta \rightarrow');
ylabel('f(x,y) \rightarrow');
```

******************************** **Figure S3** *********************************

```
clear all
set(0,'DefaultAxesFontSize',20);

alpha=1;
g=0.001;

M=100000;

x=zeros(1,M+1);
y=zeros(1,M+1);
t=zeros(1,M+1);

x(1)= 0.4;
y(1)=0.05;
t(1)=0;
dt=0.1;

%% figure;

subplot(2,2,1)
  beta=13.6;
  X1=0:0.01:1;
  Y1=(alpha-X1);
  Y2=0:0.1:1;
  X2(1:length(Y2))=(1+g*Y2)/beta;
  plot(X1,Y1,'k-');
  hold on;
  plot(X2,Y2,'r-');

for j=1:M
    t(j+1)=t(j)+dt;
    x(j+1)=x(j)+dt*(alpha*x(j)-x(j)^2-x(j)*y(j));
    y(j+1)=y(j)+dt*(beta*x(j)*y(j)-y(j)-g*y(j)^2);
end

plot(x,y,'b','MarkerSize',10, 'LineWidth',1);
```

```
xlabel('x \rightarrow');
ylabel('y \rightarrow');
X1(1:length(Y1))=(1+g*Y1)/beta;
Y1=(alpha-X1);
plot(0,0,'g.',alpha,0,'k.',X1,Y1,'g.',1,0,'g.','MarkerSize',20);


subplot(2,2,2)
  beta=13.6;
  X1=0:0.01:1;
  Y1=(alpha-X1);
  Y2=0:0.1:1;
  X2(1:length(Y2))=(1+g*Y2)/beta;
  plot(X1,Y1,'k-');
  hold on;
  plot(X2,Y2,'r-');

for j=1:M
    t(j+1)=t(j)+dt;
    x(j+1)=x(j)+dt*(alpha*x(j)-x(j)^2-x(j)*y(j));
    y(j+1)=y(j)+dt*(beta*x(j)*y(j)-y(j)-g*y(j)^2);
end

plot(t,x,'b-','LineWidth',2);
xlabel('Time \rightarrow');
ylabel('f(x,y) \rightarrow');
grid off;


subplot(2,2,3)
  beta=13.7;
  X1=0:0.01:1;
  Y1=(alpha-X1);
  Y2=0:0.1:1;
  X2(1:length(Y2))=(1+g*Y2)/beta;
  plot(X1,Y1,'k-');
  hold on;
  plot(X2,Y2,'r-');

for j=1:M
    t(j+1)=t(j)+dt;
    x(j+1)=x(j)+dt*(alpha*x(j)-x(j)^2-x(j)*y(j));
    y(j+1)=y(j)+dt*(beta*x(j)*y(j)-y(j)-g*y(j)^2);
end

plot(x,y,'b','MarkerSize',10, 'LineWidth',1);
xlabel('x \rightarrow');
ylabel('y \rightarrow');
X1(1:length(Y1))=(1+g*Y1)/beta;
Y1=(alpha-X1);
plot(0,0,'g.',alpha,0,'k.',X1,Y1,'g.',1,0,'g.','MarkerSize',20);


subplot(2,2,4)
  beta=13.7;
  X1=0:0.01:1;
  Y1=(alpha-X1);
  Y2=0:0.1:1;
  X2(1:length(Y2))=(1+g*Y2)/beta;
  plot(X1,Y1,'k-');
  hold on;
```

```matlab
  plot(X2,Y2,'r-');

for j=1:M
    t(j+1)=t(j)+dt;
    x(j+1)=x(j)+dt*(alpha*x(j)-x(j)^2-x(j)*y(j));
    y(j+1)=y(j)+dt*(beta*x(j)*y(j)-y(j)-g*y(j)^2);
end

plot(t,x,'b-','LineWidth',2);
xlabel('Time \rightarrow');
ylabel('f(x,y) \rightarrow');
grid off;
```
